# Incidence of Rotavirus and Circulating Genotypes in Northeast Brazil during 7 Years of National Rotavirus Vaccination

**DOI:** 10.1371/journal.pone.0110217

**Published:** 2014-10-31

**Authors:** Ricardo Q. Gurgel, Alberto De Juan Alvarez, Alda Rodrigues, Robergson R. Ribeiro, Sílvio S. Dolabella, Natanael L. Da Mota, Victor S. Santos, Miren Iturriza-Gomara, Nigel A. Cunliffe, Luis E. Cuevas

**Affiliations:** 1 Federal University of Sergipe, Aracaju, Brazil; 2 Liverpool School of Tropical Medicine, Liverpool, United Kingdom; 3 Institute of Infection and Global Health, University of Liverpool, Liverpool, United Kingdom; The Australian National University, Australia

## Abstract

**Background and Aims:**

Rotavirus causes severe diarrhoea and Brazil introduced the Rotarix G1P[8] vaccine in 2006. We aimed to describe changes in rotavirus incidence and diarrhoea epidemiology before and after vaccine introduction.

**Methods:**

Design: (i) hospital-based survey of children with diarrhoea (2006–2012); (ii) diarrhea-mortality and hospitalization surveillance (1999–2012).

**Setting:**

(i) Aracaju and (ii) state and national level.

**Results:**

1841 children were enrolled and 231 (12.5%) had rotavirus. Rotavirus was less frequent from January-June than from July-December (9.4% versus 20.9%, p<0.01), but the seasonal variation was less defined after 2009. Very few rotavirus cases (8–3.9%) were detected in 2011, with an increase in 2012 (13–18.5%). In 2006, unvaccinated children were more likely to have rotavirus, but thereafter unvaccinated and vaccinated children had equally low incidence. Older children and those with rotavirus were more likely to have severe diarrhea episodes. The most frequent genotype from 2006 to 2010 was G2P[4]; except in 2009, when most cases were G1P[8]. Very few G2P[4] were detected from 2011 and 50% cases in 2012 were G8P[4]. Diarrhoea-hospitalizations decreased nationally from 89,934 (2003) to 53,705 (2012; 40.3% reduction) and in the state from 1729 to 748 (56.7% reduction). Diarrhoea-deaths decreased nationally from 4368 in 1999 to 697 in 2012 (84% reduction, p<0.001) and in the state from 132 to 18 (86% reduction). These changes were much larger after vaccine introduction.

**Conclusions:**

The vaccine was associated with substantial reductions in rotavirus incidence and diarrhoea-hospitalizations and deaths. The G2P[4] genotype predominance disappeared over time and may be replaced by other heterotypic genotypes.

## Introduction

The licensing of two rotavirus vaccines in 2006 and their large scale use in countries such as Brazil and the USA marked the beginning of a new era in paediatric diarrhoeal disease control [1], [2]. Soon after their adoption, national immunisation programs reported significant reductions in diarrhoea-related hospitalisations and ambulatory consultations. In 2012 following pivotal Phase III clinical trials, the World Health Organisation (WHO) recommended extension of their use to include countries with high diarrhoea burden in Africa and Asia [3]. Vaccine introduction in these continents is rapidly gaining momentum with support from international partners including the Global Alliance for Vaccines Initiative (GAVI).

Brazil was among the first countries to integrate a rotavirus vaccine into its national immunisation programme, the monovalent G1P[8] Rotarix vaccine (Glaxo Smith Kline Biologicals) in March 2006. Although Rotarix has good efficacy for most homotypic genotypes (91.8% for G1P[8]), efficacy against fully heterotypic genotype G2P[4] is only 41% [1]. Children were offered two oral doses free of charge and vaccine coverage reached 82% by 2008 and has since remained above this level (http://datasus.saude.gov.br/). The vaccine introduction coincided with a decline in severe rotavirus-diarrhoea episodes, all-cause diarrhoea-related hospitalisations and ambulatory consultations [4], [5]. Although these reports were promising, Brazil reported a long-term decline in the incidence of childhood diarrhoea [6] and a longer period of observation was needed to elucidate the additional contribution of rotavirus vaccine to the control of childhood diarrhoea.

Early surveys following vaccine introduction described that nearly all rotavirus-diarrhoea episodes were associated with the heterotypic G2P[4] genotype [7], [8]. Although this occurrence could have been due to a temporal coincidence, as this genotype was also circulating in several Latin American countries at that time including some without rotavirus vaccination [9], it could also be due to the immunological pressure exerted by the vaccine against other genotypes for which it has higher efficacy [10]. We now report long-term changes in the epidemiology of childhood diarrhoea, rotavirus incidence and genotypes in the first seven years after vaccine introduction.

## Methods

### Ethics statement

The study protocol was approved by the research ethics committees of the Liverpool School of Tropical Medicine, Sergipe’s Federal University and the Brazilian National Commission for Ethics and Research (CONEP). Parents were requested provide written informed consent before enrolling their children in the study.

### Study design

The study comprised (i) a hospital-based survey of the proportion, severity and genotypes of acute diarrhoea episodes due to rotavirus among children attending a reference hospital in Sergipe, Northeast Brazil and (ii) analysis of thirteen-year routinely generated regional and national surveillance data comprising seven years before and six years after rotavirus vaccine introduction.

### Hospital-based survey

This was a prospective survey of children <12 years old presenting with diarrhea of <14 days duration to the pediatric emergency service of Sergipe Emergency Hospital (Hospital de Urgências de Sergipe - HUSE) from October 2006 to April 2012. HUSE is the largest reference hospital in Aracaju, the capital of Sergipe State in Northeast Brazil, containing 570,937 and 2,068,031 inhabitants, respectively [11]. Although HUSE provides 24-hour services, for logistical reasons only children attending between 8 am and 4 pm from Monday to Friday were included. We included a sample of these children to obtain a representative number of children for the month. We aimed to include a minimum of 300 specimens per year, assuming the prevalence of rotavirus would be 20% of cases per year and this sample size would allow establishing this proportion with +/−5% margin of error. After providing informed written consent, parents and children were interviewed to establish the clinical profile and vaccination history. Parents were asked to collect a stool specimen in containers before leaving the services and the approximately 60% of parents able to provide specimens were included in the final dataset. Rotavirus vaccination status was verified against the child’s vaccination card. In Brazil, most parents carry the child vaccination card because this is often requested when attending health services and it is therefore possible to confirm the vaccination status of most children. Children with two rotavirus vaccine doses recorded were considered vaccinated, while those without doses or only one dose were considered unvaccinated. Children without vaccination cards were classified as having an *unknown* vaccination status.

Stools specimens were stored at −70°C until tested using an enzyme linked immunosorbent assay (ELISA, Rotaclone; Meridian Diagnostics, Cincinnati, OH). Rotavirus genotypes were determined in ELISA-positive specimens using a hemi-nested reverse transcription-polymerase chain reaction using consensus and type-specific primers as described earlier [8].

### Surveillance data

Rotavirus vaccine coverage data were obtained for 2007–2011 from the Expanded Program of Immunization databases at http://pni.datasus.gov.br/inf_estatistica_cobertura.asp and mortality data for 1999–2012 were obtained from the national surveillance system (Datasus) available at http://www2.datasus.gov.br/DATASUS/index.php?area=0205. Data were extracted for Aracaju, Sergipe and nationally using the International Diseases Classification (IDC) codes A08 and A09. Diarrhea-related hospitalization data were obtained from www.datasus.gov.br and the Hospital-based Information System (SUS-SIH/SUS) using the IDC codes J12–J18. Data were grouped by age (<1, 1 to 4, 5 to 10 and 10 to 14 years). Frequencies for 2012 were preliminary at the time of preparing the manuscript and thus may be revised once officially published in its final form in the website.

Descriptive statistics were used to ascertain changes in the distribution of diarrhea in vaccinated and unvaccinated children, clustering of genotypes over time and their association to disease severity. Differences in proportions were tested using Chi Squares. Diarrhea severity was described using a frequently used severity score [12]. Ninety five percent confidence intervals (95% CIs) and Student’s *T* tests were used as appropriate.

## Results

### Characteristics of hospital participants

A total of 1841 children were enrolled at the HUSE survey from October 2006 to April 2012. Most children were young, with a median age of 12 months. Children in 2006–2009 were younger than children in 2010–2012 (p = 0.003– table 1) and more boys than girls were enrolled each year except in 2012. The median diarrhea duration before consultation was 3 days and the median number of stools before consultation varied from 4 to 5 episodes per day over the study period. The proportion of children vomiting was lower in 2006–2008 than in later years, increasing from 44.4% in 2007 to 73% in 2012 (p = <0.01). The diarrhea severity score was calculated for 1836 (99.7%) children and the mean severity score was 10.4, ranging from 9.2 in 2008 to 12.3 in 2012. In total 1108 (59%) children had mild/moderate (score <12) and 728 (38.9%) severe episodes (score ≥12). The proportion of children with scores ≥12 increased over the years from 27.6% in 2009 to 58.3% in 2012 (p<0.001). Diarrhea severity also increased with age, with 139/466 (29.8%) children <6 months, 160/438 (36.5%) 6−<12 months old, 178/405 (44%) 12−<24 months old and 255/531 (48%) children ≥24 months having severe episodes (p = <0.001).

**Table 1:** Characteristics of the participants with acute diarrhea from 2006 to 2012.

	2006	2007	2008	2009	2010	2011	2012	Total
Number	73	324	416	387	363	206	72	1841
Male (%)	41 (56.2%)	185 (57.1%)	219 (52.6%)	217 (56.1%)	212 (58.4%)	110 (53.4%)	32 (44.4%)	1016 (55.2%)
Age, median [range], months	12 [1–144]	10 [1–144]	12 [0–138]	11 [0–131]	13 [1–132]	16 [1–144]	14 [1–131]	12 [0–144]
Age group, months (%) <6	22 (30.1%)	113 (34.9%)	126 (30.3%)	124 (32.0%)	87 (24.0%)	49 (23.8%)	18 (25.0%)	539 (29.3%)
6−<12	18 (24.7%)	70 (21.6%)	89 (21.4%)	87 (22.5%)	90 (24.8%)	36 (17.5%)	13 (18.1%)	403 (21.9%)
12−<24	13 (17.8%)	59 (18.2%)	76 (18.3%)	76 (19.6%)	93 (25.6%)	47 (22.8%)	24 (33.3%)	388 (21.1%)
≥24	20 (27.4%)	82 (25.3%)	125 (30.0%)	100 (25.8%)	93 (25.6%)	74 (35.9%)	17 (23.6%)	511 (27.8%)
Vaccination status								
Card available	73 (100%)	196 (60.5%)	204 (49.0%)	297 (76.7%)	259 (71.3%)	162 (78.6%)	60 (83.0%)	1451 (78.8%)
*Vaccinated	21 (28.8%)	103 (52.6%)	130 (63.7%)	184 (62.0%)	197 (76%)	117 (72.2%)	52 (86.7%)	804 (55.4%)
Not vaccinated	52 (71.2%)	93 (47.4%)	74 (36.3%)	113 (38.0%)	62 (24%)	45 (27.8%)	8 (13.3%)	647 (44.6%)
Diarrhoea								
**Median duration [range]	3 [1]–[13]	3 [1]–[15]	3 [0–60]	3 [1]–[17]	3 [0–30]	3 [1]–[15]	3 [1]–[15]	3 [1]–[15]
Frequency per day	5 [3]–[15]	5 [3]–[20]	4 [1]–[20]	4 [1]–[15]	4 [1]–[25]	5 [1]–[30]	5 [2]–[16]	4 [1]–[30]
Vomiting								
Present	41 (56.2%)	144 (44.4%)	230 (55.3%)	258 (66.7%)	244 (67.2%)	152 (73.8%)	53 (73.6%)	1122 (60.9%)
Median duration [range]**	1 [0–8]	0 [0–15]	1 [0–10]	1 [0–9]	1 [0–63]	1 [0–6]	1 [0–10]	1 [0–63]
Frequency per day	1 [0–15]	0 [0–15]	1 [0–20]	1 {0–15]	1 [0–20]	1 [0–30]	2 [0–18]	1 [0–30]
Severity score								
Mean	11.3	9.7	9.2	10.6	10.9	11.5	12.3	10.4
Score ≥12 (%)	33 (45.2%)	112 (34.6%)	115 (27.6%)	157 (40.6%)	158 (43.0%)	114 (54.9%)	43 (58.3%)	732 (39.6%)
Rotavirus ELISA positive (%)	18 (24.7%)	30 (9.3%)	77 (18.5%)	41 (10.6%)	43 (11.8%)	8 (3.9%)	14 (19.4%)	231 (12.5%)

*Only includes children with vaccination cards; **in days.

Vaccination cards were available for 1451 (79%) children and rotavirus vaccines were recorded in 28.8% participants in 2006 and 86.7% in 2012, with an expected increase over time (p<0.001).

**Figure 1:**
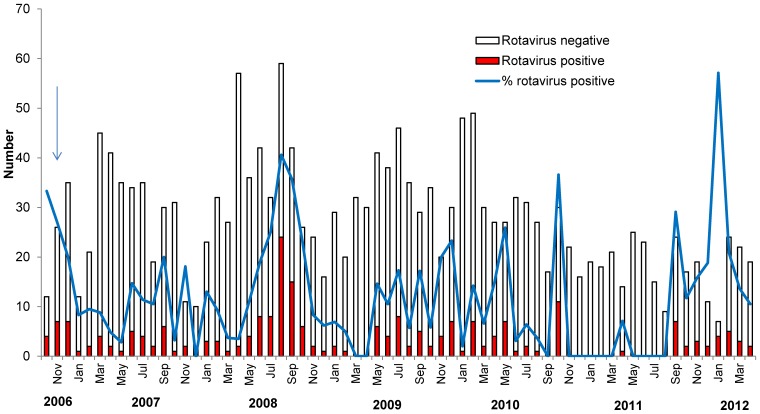
Number of children enrolled and proportion with rotavirus infection by month, November 2006 to April 2012. The arrow illustrates the date of vaccine introduction.

Two-hundred and thirty one (12.5%) children were rotavirus-ELISA positive. The proportion of children with positive ELISAs varied by month and was lower during the months of January to June and higher from July to December (86/914 [9.4%] versus 145/695 [20.9%], respectively, p<0.01). This seasonal variation became less well defined since 2009 and very few rotavirus cases were detected in 2011 (Figure 1). The 2011 nadir however was then followed by an increase in the number of cases in 2012, when a much higher percentage of children had rotavirus.

**Figure 2:**
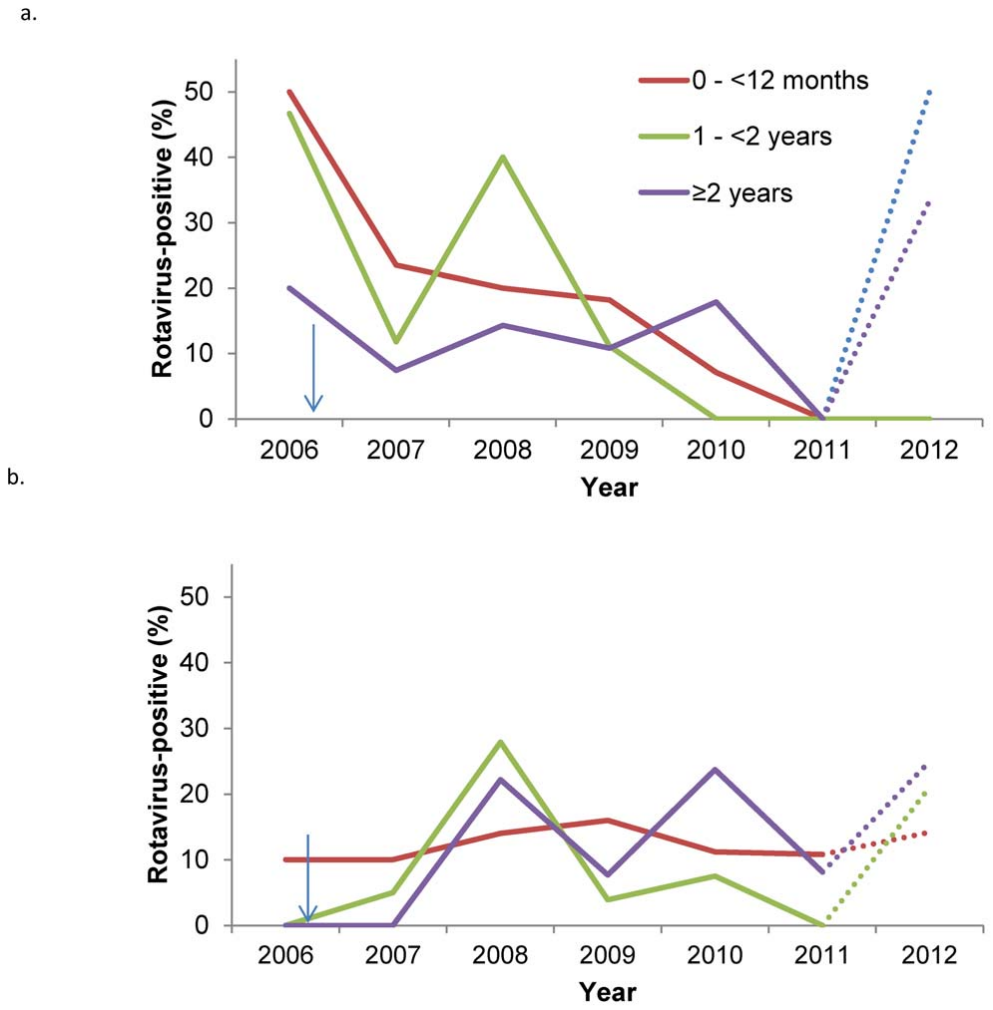
Proportion of (a) non-vaccinated and (b) vaccinated children attending the hospital with rotavirus, by age and year. Percentages for 2012 are incomplete (to March, represented by a dotted line). Arrows illustrate the vaccine introduction.

**Figure 3:**
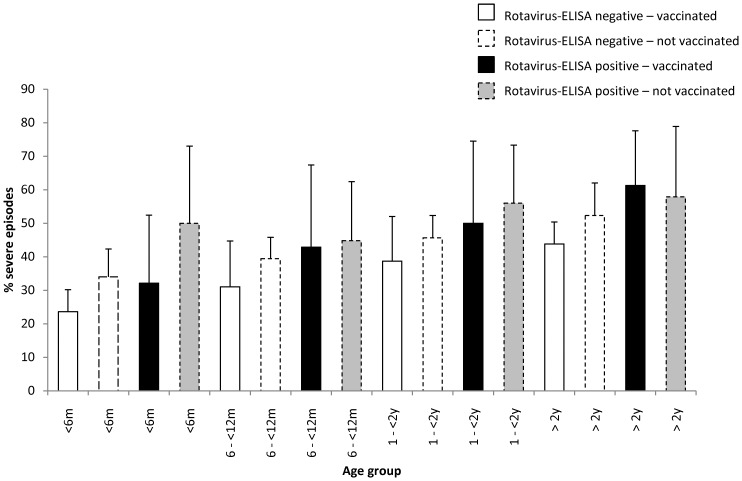
Proportion of vaccinated and non-vaccinated children attending the Emergency service with severe diarrhea, by age and presence of rotavirus.

The proportion of children with rotavirus by age and vaccination status is shown in figure 2. In 2006, unvaccinated children <24 months were more likely to have rotavirus than unvaccinated children ≥24 months old. Vaccinated children were also less likely to have rotavirus than unvaccinated children. This pattern changed after 2006, as a low percentage of both vaccinated and unvaccinated children had rotavirus. The change was observed in all age groups. The proportion of children with severe episodes increased with age in both children with and without rotavirus (p<0.001 for both) and children with rotavirus had more severe episodes than children without rotavirus, as shown in Figure 3. Generally, vaccinated children had less severe episodes than unvaccinated children, but this difference was only statistically significant in children <6 months.

Two-hundred twenty seven rotavirus-ELISA positive stools underwent G and P genotyping. Five G and three P types were identified over the six years, as shown in Table 2. The most prevalent G types were G2 (166, 73%), G1 (40, 17.6%) and G8 (13, 5.7%). Other G types identified included G3 (3, 1.5%), G12 (2, 0.9%) and four G non-typeable. The most frequent P types were P[4] (179, 78.9%), P[8] (35, 15.4%) and P[6] (8, 3.5%), and 10 were non-typeable. The most common G and P combinations were G2P[4] (160, 70.5%), G1P[8] (29, 12.7%), G8P[4] (12, 5.3%) and G3P[8] (3, 1.3%). Less frequent combinations included G1P[4] and G1P[6], G2P[6], G8P[6] and G12P[4] and G12P[8]. Five stools had more than one genotype, including two G2P[4]P[8] and one each of G2G8P[1], G1G2P[4], with and G8G2P[4]. The frequency of the genotypes varied over time. G2P[4] was the only genotype identified from 2006 to 2009. Then in 2009, the majority of rotaviruses were G1P[8], with G2P[4] having a lower frequency. In 2010, G2P[4] was again the predominant genotype and other genotypes had lower frequencies. A variety of genotypes were identified in 2011 but with low frequencies, while seven of 14 isolates in 2012 belonged to the uncommon G8P[4] genotype.

**Table 2:** Genotypes identified in children attending the emergency hospital with diarrhea (2006–2012).

	ELISA-positive/tested	Genotype	N	(%)
**2006**	18/73 (25%)	G2P[4]	16	(89)
		GNTP4	1	(6)
		G2PNT	1	(6)
**2007**	30/324 (9.3%)	G2P[4]	28	(93)
		GNTPNT	2	(7)
**2008**	77/416 (18.5%)	G2P[4]	77	(100)
**2009**	41/384 (10.7%)	G1P[8]	28	(68)
		G2P[4]	9	(22)
		G2P[4]P[8]	1	(2)
		G2PNT	2	(5)
		GNTPNT	1	(2)
**2010**	40/335 (11.9%)	G2P[4]	26	(65)
		G1P[6]	4	(10)
		G1P[4]	3	(8)
		G2P[6]	2	(5)
		G2P[4]P[8]	1	(3)
		G1P[8]	1	(3)
		G8P[4]	1	(3)
		G12P[8]	1	(3)
		G12P[4]	1	(3)
**2011**	8/207 (3.9%)	G2P4	2	(25)
		G3P[8]	2	(25)
		G8P[4]	2	(25)
		G2G8P[4]	1	(13)
		G2PNT	1	(13)
**2012**	13/70 (18.5%)	G8P[4]	7	(54)
		G8P[6]	2	(15)
		G1G2P[4]	2	(15)
		G3P[8]	1	(8)
		G8G2P[4]	1	(8)
**All**	227/1809 (12.5%)			

### Surveillance data

**Figure 4:**
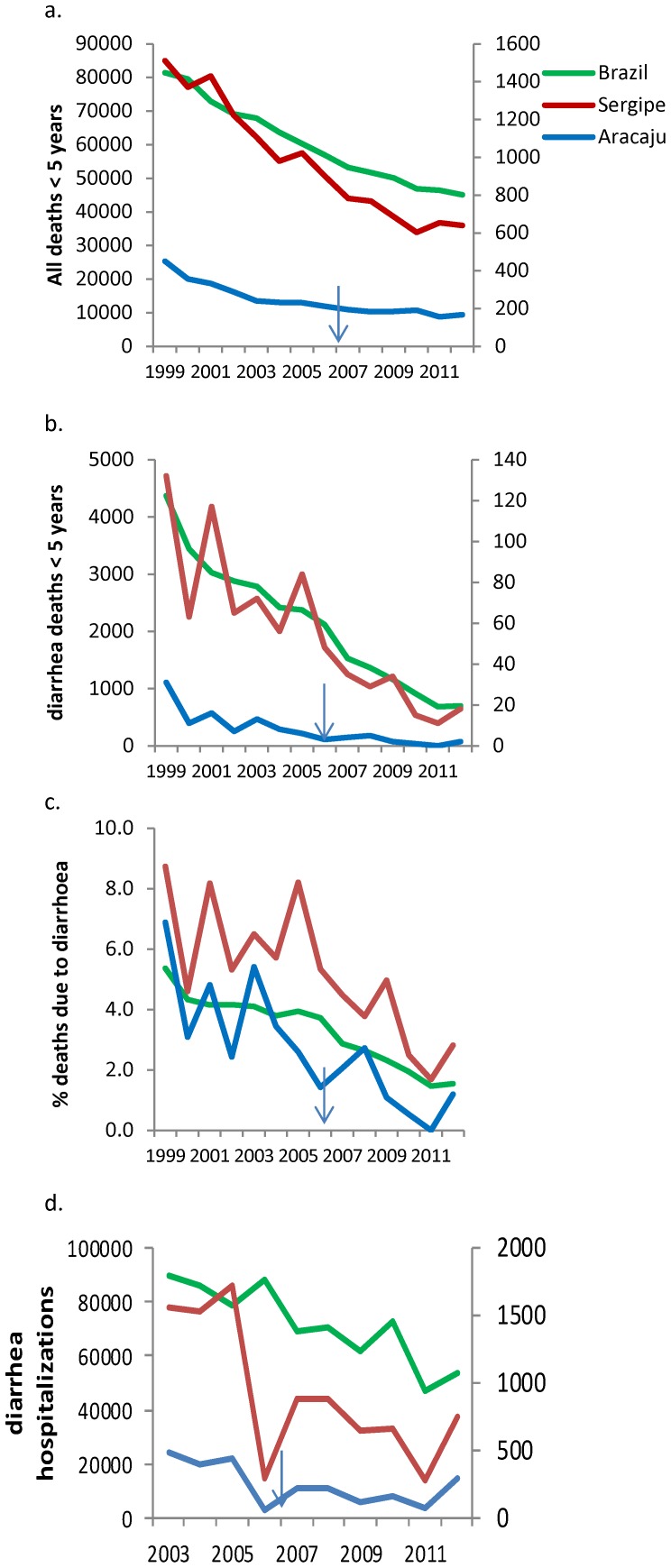
Childhood (<5 years old) all cause and diarrhea deaths and hospitalizations in Brazil, Sergipe and Aracaju City. Arrows illustrate the year of vaccine introduction.

All cause and diarrhoea-deaths occurring in children <5 years old in Brazil, Sergipe State and Aracaju from 1999 to 2012 are shown in Figure 4 (a) and (b). All-cause deaths decreased nationally from 81,391 deaths in 1999 to 45,101 in 2012 (45% reduction from the 1999 baseline). Sergipe State recorded 1,511 in 1999 and 639 in 2012 (58% reduction) and Aracaju had the largest percentage reduction with 450 deaths in 1999 and 167 in 2012 (63% reduction). The reduction trends for all-cause deaths were similar between 1999–2005 and 2006–2012 nationally and at state and city level.

Diarrhoea-specific deaths decreased more sharply than all-cause deaths, with a national reduction from 4368 deaths in 1999 and 697 in 2012 (84% reduction) (p<0.001). Sergipe State reported similar reductions, decreasing from 132 deaths in 1999 to 18 in 2012 (86% reduction). Aracaju city reported similar reductions, from 11 deaths in 1999 to two in 2012 (82% reduction). Percentage reductions in diarrhoea deaths were larger than for all-cause deaths (p<0.001 for national, state and city level). The decreasing trend for diarrhoea deaths were also different before and after 2006 at the national level, with a 45% reduction from 1999 to 2005 and an additional 71% reduction from 2006 to 2012 (p<0.001). Sergipe reported a 36.4% reduction between 1999 and 2005 and 78% reduction from 2006 to 2012 (p<0.001). Diarrhoea deaths therefore decreased more rapidly than all-cause deaths. The proportion of all-cause deaths due to diarrhoea changed nationally from 5.4% in 1999 to 1.5% in 2012. Similarly in Sergipe and Aracaju, diarrhoea deaths represented 8.7% and 6.9% of all deaths in 1999, but only 2.8% and 1.2% in 2012 (Figure 4c).

Diarrhoea-related hospitalisations in Brazil, Sergipe and Aracaju from 2003 to 2012 are shown in Figure 4d. Diarrhoea hospitalizations decreased nationally from 89,934 in 2003 to 53,705 in 2012 (40.3% reduction). The percentage decrease was also large in Sergipe and Aracaju, decreasing from 1729 to 748 (56.7% reduction) in Sergipe and from 483 to 296 (38.7% reduction) in Aracaju. The reduction in the number of diarrhoea-hospitalizations varied before and after vaccine introduction, with a 1.7% reduction in the national number of hospitalizations from 2003 to 2005 and a 39.2% reduction between 2006 and 2012. Similarly Sergipe State reported a 10% increase in diarrhoea hospitalizations between 2003 and 2005 but a 56.7% reduction between 2006 and 2012 (p<0.001).

## Discussion

Diarrhoea is the second most important cause of child death globally, with most cases occurring in low and middle income countries and rotavirus is the pathogen most frequently associated with severe episodes [13], incurring substantial hospitalisation, medical consultations and parental costs [14]. The advent of rotavirus vaccines and their rapid adoption by national immunisation programmes heralded a new era of vaccine-based interventions for diarrhoea control [1], [2] with enormous potential for health benefit. Brazil’s adoption of a rotavirus vaccine created one of the largest rotavirus-vaccinated cohorts ever and monitoring changes in the burden of rotavirus and all cause-diarrhoea in the country could generate information for countries considering the adoption of the vaccines.

This study demonstrates that Brazil experienced major epidemiological changes in rotavirus-specific and all cause diarrhoea. Surveillance data confirm the long term decline in all cause and diarrhoea-specific childhood mortality [6], [15], which have largely been attributed to improved accessibility to services, sanitation and public health interventions [15]. These trends are considerable confounders to assess changes occurring after vaccine introduction. We therefore have described whether the secular trends changed before and after vaccine introduction, and whether the proportion of all deaths that are due to diarrhoea changed before and after vaccine introduction. Both indicators suggest that the vaccine introduction was associated with statistically significant changes in the trends. Not only the number of diarrhoea deaths decreased more rapidly after vaccine introduction, but the proportion of diarrhoea became a smaller proportion of all deaths. Nationally, the proportion of deaths attributed to diarrhoea declined from 5.4% of all deaths in 1999 to 3.9% by 2005, but changed again from 3.9% in 2005 to 1.5% in 2012. The same pattern was observed for Sergipe, with a 36% reduction in the number of episodes between 1999 and 2005 and a 78% reduction after vaccine introduction, with the proportion of childhood deaths due to diarrhoea reducing from 8.2% in 2005 to 2.5% in 2012. The patterns therefore strongly suggest that these changes are due to the vaccine and the higher proportion of diarrhoea deaths in Sergipe is in agreement with previous data reporting a higher diarrhoea mortality in the Northeast of the country[6].

Data for diarrhoea-related hospitalizations were only available from 2002 and secular trends before vaccine introduction were less well documented. However the data suggests that changes in the number of hospitalizations before vaccine introduction were minimal. These trends had an evident change after vaccine introduction, with a 39.2% national reduction in diarrhoea-related hospitalization and an even larger 56.7% reduction in Sergipe. These data thus confirm that the vaccine is associated with significant reductions in hospitalizations and that these reductions were higher in a state where diarrhoea was a significant public health problem. Reductions in diarrhoea-related deaths and hospitalisations have been reported from Latin America [16], the United States [17], Europe [18], [19] and Australasia [20] and interestingly these reductions have been higher than expected from previous impact models, which has been attributed to many cases being undetected before vaccine introduction and a sustained vaccine efficacy over several years [21].

Our hospital data show that the proportion of children with rotavirus infection has also decreased over the years, with a remarkable low proportion of cases having rotavirus in 2011. This low proportion was observed in both vaccinated and unvaccinated children and in infants and older children. Although counterintuitive, our findings are in agreement with reports demonstrating the significant herd protection of the vaccine [22], [23], which is likely due to the reduced virus transmission in the environment and possibly vaccine strains spreading to unvaccinated children [24].

Our study also documents changes in the rotavirus genotypes. A high proportion of G2P[4] strains from 2006 to 2010 had been reported by us and others in Brazil and by countries that use the Rotarix vaccine in Australasia [20] and Europe [19], [22], [25]. We have however also observed further changes in 2011, when the G2P[4] became less frequent and other strains, such as G8[4], G8P[6] and G3P[8)] became more frequent. The G2P[4] decrease and the increase of other unusual G8 strains suggest that these changes may not be limited to the G2P[4] genotype. G8 is considered to have a bovine origin and is not included in the current vaccines [26] and has frequently been reported from African countries, where it accounts for about 12% of cases [27] and has recently been reported from Europe [28]. A further genotype increasingly isolated in 2011/12 was G3P[8]. This genotype has been reported with increased frequency in regions with a high coverage of RotaTeq [10], but it is also one of the five globally common rotavirus genotypes and its significance in the Brazilian context needs further study.

It is also interesting that despite the reduced mortality and hospitalizations, the proportion of diarrhea episodes classified as severe has increased over the years. This increase occurred both in vaccinated and unvaccinated children and in children with and without rotavirus. This might be an artefact caused by self-selection of patients, changes in service organization and provision and older children becoming more prominent because younger children are protected by the vaccine. In addition, other pathogens not included in this analysis (e.g. norovirus) may become more established and further studies are needed to establish if other pathogens may take the ecological niche of rotavirus.

Our study also has important limitations. The evaluation was partly conducted using data collected through surveillance systems, which are not devoid of problems. Surveillance is often based on sentinel sites that may change over time and become unrepresentative and incomplete data reporting may underestimate the mortality and hospitalizations attributed to diarrhoea. Furthermore, using all-cause diarrhoea as a marker for rotavirus diarrhoea could mask changes in the epidemiology of other pathogens. In addition, the hospital case series only recorded a child as vaccinated if both doses of the vaccine were recorded, and not vaccinated if the child had received none or one vaccine dose. Labelling partially vaccinated children as unvaccinated could have reduced the number of episodes in the non-vaccinated group. However rotavirus infections were uncommon in all children and very few of the children receive only one dose of the vaccine, and for these reasons we believe this limitation is unlikely to modify the interpretation of the data. Furthermore, children admitted to the hospital outside working hours were assessed the following morning, but ambulatory children attending at night were missed. If attendance patterns and genotype distributions were associated with clinical severity, then this sampling strategy could have led to bias towards children with mild or severe presentations and the exclusion of children with moderate diarrhea and distortions in the genotype distribution.

Despite these shortcomings, similar approaches are being used to monitor vaccine efficacy elsewhere and African countries are preparing to use this method to monitor the vaccines when introduced on a large scale in the continent [29]–[31].

A further limitation is that our prospective data collection was based in one medical centre, receiving both self-selected and referred cases and therefore genotype representation is likely to be biased towards children with more severe episodes.

Despite these limitations, the data presented here add to the evidence of the growing public health impact of rotavirus vaccines in Brazil, with large reductions in the number of diarrhoea related childhood hospitalizations and deaths. Our data also provide new evidence that the predominance of the G2P[4] genotype in countries using the monovalent rotavirus vaccine is temporal and that other unusual genotypes may appear with time. Continued surveillance therefore is needed to document the continued effectiveness of the vaccines and the potential emergence of unusual rotavirus genotypes.
